# TRPM4-specific blocking antibody attenuates reperfusion injury in a rat model of stroke

**DOI:** 10.1007/s00424-019-02326-8

**Published:** 2019-10-29

**Authors:** Bo Chen, Yahui Gao, Shunhui Wei, See Wee Low, Gandi Ng, Dejie Yu, Tian Ming Tu, Tuck Wah Soong, Bernd Nilius, Ping Liao

**Affiliations:** 1 Calcium Signalling Laboratory, Department of Research, National Neuroscience Institute, 11 Jalan Tan Tock Seng, Singapore, Singapore; 2 Department of Physiology, Yong Loo Lin School of Medicine, National University of Singapore, Singapore, Singapore; 3 Department of Neurology, National Neuroscience Institute, Singapore, Singapore; 4 Department of Cellular and Molecular Medicine, KU Leuven, Leuven, Belgium; 5 Duke-NUS Medical School, Singapore, Singapore; 6 Health and Social Sciences, Singapore Institute of Technology, Singapore, Singapore

**Keywords:** Ischemic stroke, TRPM4, Blood–brain barrier, Reperfusion injury, Time window

## Abstract

Reperfusion therapy is currently the gold standard treatment for acute ischemic stroke. However, reperfusion injuries such as oedema and haemorrhagic transformation largely limit the use of this potent treatment to a narrow time window. Recently, transient receptor potential melastatin 4 (TRPM4) channel has emerged as a potential target for vascular protection in stroke management. Non-specificity and side effects are major concerns for current TRPM4 blockers. The present study was undertaken to develop a novel TRPM4 blocker for stroke management. We report the generation of a TRPM4-specific antibody M4P which binds to a region close to the channel pore. M4P could inhibit TRPM4 current and downregulate TRPM4 surface expression, therefore prevent hypoxia-induced cell swelling. In the rat model of 3-h stroke reperfusion, application of M4P at 2 h after occlusion ameliorated reperfusion injury by improving blood–brain barrier integrity, and enhanced functional recovery. Our results demonstrate that TRPM4 blockade could attenuate reperfusion injury in stroke recanalization. When applied together with reperfusion treatments, TRPM4 blocking antibody has the potential to extend the therapeutic time window for acute ischemic stroke.

**Electronic supplementary material:**

The online version of this article (10.1007/s00424-019-02326-8) contains supplementary material, which is available to authorized users.

## Introduction

Transient receptor potential melastatin 4 (TRPM4) is a voltage-dependent, nonselective monovalent cation channel which is activated by elevated cytosolic Ca^2+^, and modulated by ATP depletion [1]. Under hypoxic conditions, its expression is upregulated, and channel activities are enhanced by oxygen depletion, leading to oncotic cell death [2]. Therefore, TRPM4 inhibition could alleviate oedema formation by stabilising the blood–brain barrier (BBB) [3]. Recently, TRPM4 has emerged as a therapeutic target for many brain disorders such as stroke, spinal cord injury, and head injury.

TRPM4 has been reported to interact with sulfonylurea receptor-1 (Sur1) to form a SUR1-TRPM4 channel complex, and application of SUR1 blockers sulphonylureas could inhibit SUR1-TRPM4 function [4]. SUR1 is an auxiliary subunit of K_ATP_ channel which senses ATP changes in pancreatic β cells, and regulates insulin secretion [5]. Therefore, sulphonylureas such as glibenclamide are widely used to control blood glucose level in diabetic patients. As sulphonylureas are being used in clinical practice, multiple studies on stroke patients with or without diabetes mellitus were carried out using glibenclamide. The result showed that use of sulphonylureas before or after stroke onset could reduce haemorrhage transformation, attenuate cerebral oedema, and improve neurological outcome [6]. In contrast, other studies on diabetic patients who later developed stroke revealed that application of sulphonylureas achieved no better outcome than other anti-diabetic treatments [7, 8]. Such controversies may arise from differences in patient inclusion criteria, dose of sulphonylureas, or the severity of diabetes mellitus. Interestingly, a study showed that the application of sulfonylurea glimepiride achieved neuroprotection against stroke only in normal mice but not in type 2 diabetic mice [9], suggesting that the presence of diabetes mellitus could be a confounding factor for the use of sulphonylureas to manage stroke. Recently, a phase II multicentre clinical trial using glibenclamide in patients with large anterior circulation hemispheric infarction was reported [10], and there was no difference between the glibenclamide and control groups for the primary outcome, even though signs of oedema alleviation were observed. One possible reason is that the dose of glibenclamide used was low in the study, as a high dose could induce persistent hypoglycaemia in patients [11].

In view of these issues, we seek to block TRPM4 directly without targeting SUR1. The expression of TRPM4 can be inhibited at the mRNA level with gene-specific siRNA. TRPM4 siRNA could enhance vascular integrity and improve motor functions in both permanent and transient middle cerebral artery occlusion (MCAO) models [12, 13]. As siRNA functions at the mRNA level, the time of application is critical. Once TRPM4 protein is upregulated, the therapeutic effect of siRNA becomes less effective. In this study, we describe the production of a TRPM4-specific blocking antibody M4P and demonstrate that M4P could improve stroke outcome in stroke reperfusion model. As M4P does not interact with SUR1/K_ATP_ channel complex, potential side effects from glibenclamide can be avoided.

## Material and methods

### Generation of polyclonal antibody M4P

**Fig. 1:**
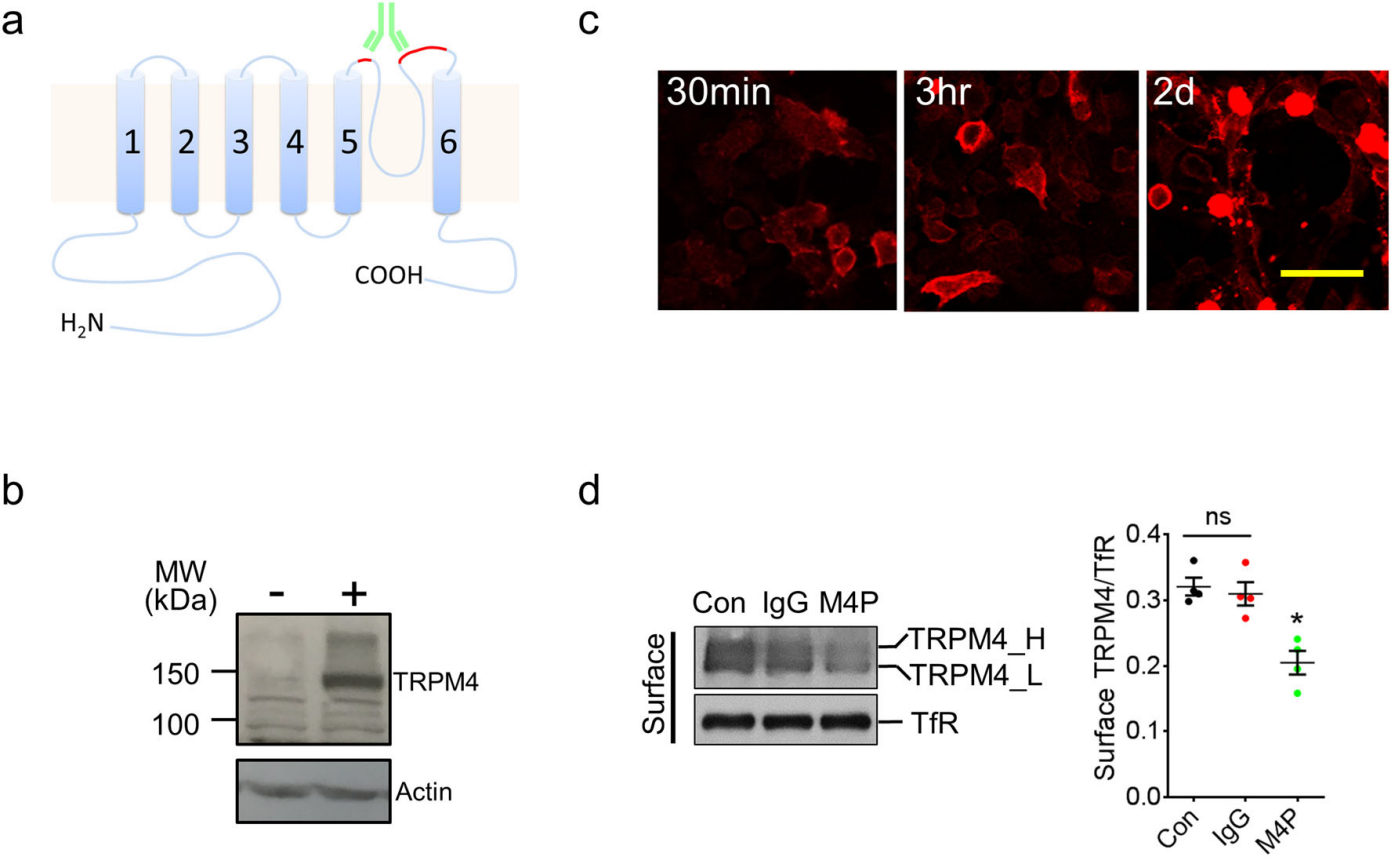
Generation and characterisation of TRPM4 blocking antibody M4P. **a** Schematic representation of TRPM4 channel with the antigenic epitope for M4P labelled in red. **b** Using M4P to detect TRPM4 channel in HEK 293 cells transfected with mouse TRPM4 (+) or an empty vector (−). **c** Live HEK 293 cells with TRPM4 expression were incubated with 1.3 μg/ml M4P for 30 min, 3 h and 2 days, and fixed for immunostaining to detect M4P localization. Scale bar 50 μm. **d** Detection and quantification of TRPM4 channel expression. TRPM4 transfected HEK 293 cells were incubated with rabbit IgG (1.3 μg/ml) and M4P (1.3 μg/ml) for 6 h, then surface biotinylation assay was performed. Con: control group received no treatment. For quantification, *n* = 4 experiments, and statistical analysis was performed by one-way ANOVA with Bonferroni’s post hoc analysis. *p < 0.05, M4P vs Con or IgG. No significance was detected between Con and IgG (ns)

The procedure to generate rabbit polyclonal antibody M4P is similar to our previous report [13, 14]. In brief, DNA sequence encoding rat TRPM4 polypeptide antigen (Fig. 1a in the online-only Data Supplement) was cloned in frame into pGEX-4T-1 plasmid. GST-fused protein was purified with glutathione-agarose (G4510, Sigma-Aldrich, MI, USA) (Fig. 1c in the online-only Data Supplement). 0.5 mg purified protein was injected into female New Zealand White rabbits subcutaneously once a month. Complete Freund’s adjuvant was used for the first immunisation, and incomplete Freund’s adjuvant was used in subsequent injections. Ten millilitre of blood was collected from the rabbit ear vein every month for serum extraction. To produce purified M4P, 0.5 ml beads containing GST protein was used to eliminate nonspecific antibodies against GST. The serum was then incubated with PVDF membrane containing 1 mg immobilised antigen overnight at 4 °C before affinity-purified with an IgG elution buffer (21004, Thermo Fisher Scientific, MA, USA). The purified M4P antibody was quantified and diluted to 1 μg/μl for experiment.

### Animal model and study design

This study was approved and conducted in accordance with the guidelines of the Institutional Animal Care and Use Committee of the National Neuroscience Institute, Singapore. All experiments were performed according to Stroke Therapy Academic Industry Roundtable (STAIR) recommendations [15]. Allocation of animal treatment was randomised by throwing a dice. The middle cerebral artery occlusion (MCAO) method has been described previously [13].

The animals were housed with temperature maintained at around 23 °C and 12/12-h light/dark cycle was set. Pelleted food and water were available for the animals. The animals were monitored on a daily basis. All researchers involved in the study were blinded to the intervention. The infarct volume calculation at day 1 post operation was a predetermined primary end point, and the completion of functional study was the secondary end point. The study would be terminated if the mortality rate at primary end point is more than 60%. The mortality rates from different treatments were calculated and compared. Animal death during operation was not counted for mortality analysis. Animal exclusion criteria include (1) cerebral blood flow reduction post occlusion was < 50%; (2) rats without motor functional deficit assessed by Rotarod test (≥ 100% of baseline); (3) rats died during the observation periods.

In brief, male Wistar rats weighing approximately 250–280 g were anesthetised with ketamine (75 mg/kg) and xylazine (10 mg/kg) intraperitoneally. Relative regional cerebral blood flow of the animals was monitored by a Laser-Doppler flowmetry (moorVMS-LDF2™, Moor Instruments Inc., DE, USA). Heart rate, blood pressure, and rectal temperature were monitored using a data acquisition system PowerLab 4/35 from AD Instruments. The body temperature was maintained at 37 °C ± 0.5 °C with a warm pad throughout the procedure. The left common carotid artery (CCA), internal carotid artery (ICA), and external carotid artery (ECA) were dissected out. A silicon-coated filament (0.37 mm, Cat #403756PK10, Doccol Corp, CA, USA) was introduced into the left ICA through ECA. Cerebral blood flow of the animals was monitored by a Laser-Doppler flowmetry (moorVMS-LDF2™, Moor Instruments Inc., DE, USA). Animals with ≤ 50% cerebral blood flow reduction were excluded from the study. Reperfusion was achieved by removing the filament gently from the ECA at 3 h following occlusion. A single dose of 100 μg of M4P antibody [14] or control rabbit IgG (I5006, Sigma-Aldrich, MI, USA) was injected intravenously via tail vein at 2 h after occlusion (1 h before recanalization). For control permanent MCAO model, animals received similar operational procedure except for filament removal. For in vivo binding of M4P (Figs. 4a and 5a), MCAO was performed as per described. Two hours after occlusion, 100 μg of M4P antibody, control rabbit IgG, or 100 μl vehicle solution was injected intravenously. Three hours after occlusion (1 h after antibody injection), the rats were sacrificed and perfused with PBS to remove residual antibodies in the circulation. The brains were sectioned and fixed with 4% paraformaldehyde. Immunofluorescent staining using secondary antibody against rabbit IgG was performed to detect antibody extravasation.

### Infarct volume measurement

Twenty-four hours after surgery, the animals were sacrificed, and the brains were collected with cerebellum and overlying membranes being removed. The brains were sectioned into 8 slices using a brain-sectioning block, each with 2 mm in thickness. The brain slices were incubated for 30 min in a 0.1% solution of 2,3,4-triphenyltetrazolium chloride (TTC) (T4375, Sigma-Aldrich, MI, USA) at 37 °C. The sections were scanned, and the infarct size was analysed using an image analyser system (HP Scanjet G3110, HP Inc, CA, USA). Calculation of oedema-corrected lesion was performed as described previously [16].

### Motor functions

Rotarod (Ugo Basile, Gemonio, Italy) was used to evaluate motor functions post stroke. Before operation, the rats received 3 training trials with 15-min intervals for 5 consecutive days. The rotarod was set to accelerate from 4 to 80 rpm within 10 min. The mean duration of time that the animals remained on the device 1 day before MCAO was recorded as internal baseline control. At different time points following surgery, the mean duration of latency was recorded and compared.

### Cell transfection and cell death, immunofluorescent staining, western blot, and surface biotinylation

For cell culture, HEK 293 cells were seeded on coated coverslips in 35 mm petri dish. Mouse TRPM4 (pIRES-EGFP-TRPM4) was transiently expressed using the calcium phosphate transfection method. Twenty-four hours after transfection, M4P or control rabbit IgG was added into the culture medium to a concentration of 0.26 μg/ml. After incubation for 30 min, 3 h, or 2 days, the cells were fixed with 4% paraformaldehyde. After washing three times with washing buffer (0.2% Triton X-100 phosphate-buffered saline), the samples were incubated with secondary antibody against rabbit IgG (Alexa Fluor 594 conjugated, Thermo Fisher Scientific, MA, USA) for 1 h before being mounted with FluorSave^TM^ reagent (345789, Millipore, MA, USA). For rat brain staining, the brains were harvested and sectioned at 10 μm in thickness. Following fixation with 4% paraformaldehyde, the brain slice was incubated in 100 μl blocking serum (10% fetal bovine serum in 0.2% PBST) for 1 h. Primary antibodies include M4P (rabbit, 10 ng/μl), anti-NeuN (MAB377, Millipore, MA, USA, 1:250), anti-GFAP (IF03L, Millipore, MA, USA, 1:200), and anti-vWF (AB7356, Millipore, MA, USA, 1:200). Secondary antibodies are conjugated with FITC or Alexa Fluor 594. Images were visualised by a confocal microscope (Fluoview BX61, Olympus, Tokyo, Japan). ImageJ was used to quantify the fluorescent intensity and vascular diameter. The average diameter was determined by using the shortest Feret diameter (Feret Min) as described previously [17]. To quantify the fluorescence of M4P labelled vessels in rat brains, an outline was drawn around each vessel. Area, mean fluorescence, along with several adjunct background readings were measured using ImageJ. The total corrected fluorescence was calculated according to the formula: total corrected fluorescence density = (integrated fluorescence signal of selected vessels − area of selected vessels × mean fluorescence of background signal) / area of selected vessels [18, 19]. Identical acquisition conditions were used to capture images.

Cell death was determined using the Trypan blue exclusion method. TRPM4 transfected HEK 293 cells which were subjected to oxygen/glucose deprivation (OGD) for 24 h. Hypoxia was induced by culturing the cells in a hypoxic chamber with 1% O_2_ and 5% CO_2_ at 37 °C, and glucose was removed from the culture media. IgG or M4P was added to the cells at a concentration of 1.3 μg/ml before OGD induction.

For western blot on rat brains, tissues from ipsilateral and contralateral hemispheres were harvested. Infarct area which was negatively stained by TTC was excluded. For western blot on HEK 293 cells, the cells grown in 6-well petri dishes were transfected with 3 μg mouse pIRES-EGFP-TRPM4, TRPM5, or GFP vector. To perform western blot, 30 μg of total protein was resolved on 10% SDS-PAGE gels at 80 V, and electrophoretically transferred to PVDF membranes (1620177, Bio-Rad, CA, USA) at 100 V for 2 h at 4 °C. After blocking with StartingBlock (PBS) blocking buffer (37538, Thermo Fisher Scientific, MA, USA) for 1 h at room temperature, membranes were incubated overnight at 4 °C with primary antibodies: M4P (1:200), anti-GFP (12603500, Roche, Basel, Switzerland, 1:500), anti-TRPM5 (acc-045, Alomone, Jerusalem, Israel, 1:200), anti-Transferrin Receptor (TfR, 13–6800, Thermo Fisher Scientific, MA, USA, 1:1000), and anti-actin (A1978, Sigma-Aldrich, MI, USA, 1:5000). After washing away primary antibodies, the membranes were incubated with secondary antibodies (A4416, A4914, Sigma-Aldrich, MI, USA, 1:5000) for 1 h at room temperature. For western blot to detect antibody extravasation (Fig. 5b), secondary antibody against rabbit IgG was applied directly without incubation with primary antibodies. The primary antibodies were the control rabbit IgG or the polyclonal M4P injected into the rats. Primary and secondary antibodies were prepared in StartingBlock (PBS) blocking buffer with 0.05% Tween®20 (P7949, Sigma-Aldrich, MI, USA). Washing buffers contained 0.1% Tween®20 dissolved in PBS. Amersham ECL Western Blotting Analysis System (RPN2109, GE Healthcare, IL, USA) was used and the bands were visualised using medical X-ray processor (MXP-2000, KODAK, NY, USA). Quantification was done using ImageJ.

TRPM4 surface expression was characterised using an EZ-Link^TM^ Sulfo-NHS-Biotinylation Kit (Thermo Fisher Scientific, MA, USA) with slight modification [20]. HEK 293 cells transfected with TRPM4 were incubated with control rabbit IgG or M4P of 1.3 μg/ml for 6 h. The cells were then treated with 0.25 mg/ml Biotin and shaken for 1 h at 4 °C. Unbound biotin was removed by incubation with quenching buffer for 20 min and washed with cold TBS. Protein concentration of cell lysates were measured with Pierce BCA Protein Assay Kit (23227, Thermo Fisher Scientific, MA, USA). Ten microlitre of cell lysates was kept for SDS-PAGE analysis. The remaining cell lysates were incubated with NeutrAvidin (Thermo Fisher Scientific, MA, USA) overnight at 4 °C to pull down the biotinylated surface proteins. The precipitates were boiled in 2× loading buffer to elute Avidin-bound for SDS-PAGE analysis.

### Electrophysiology

Whole-cell patch clamp was used to measure TRPM4 currents in HEK293 cells grown in 24-well plates and transfected with 1 μg pIRES-EGFP-TRPM4 encoding mouse TRPM4 channel. TRPM4 currents were recorded at room temperature 24–48 h after transfection. Patch electrodes were pulled using a Flaming/Brown micropipette puller (P-1000, Sutter Instrument, CA, USA) and polished with a microforge (MF-200, WPI Inc. FL, USA). Whole-cell currents were recorded using a patch clamp amplifier (Multiclamp 700B equipped with Digidata 1440A, Molecular Devices, CA, USA). The bath solution contained (in millimole/litre) NaCl 140, CaCl_2_ 2, KCl 2, MgCl_2_ 1, glucose 20, and HEPES 20 at pH 7.4. The internal solution contained (in millimole/litre) CsCl 156, MgCl_2_ 1, EGTA 10, and HEPES 10 at pH 7.2 adjusted with CsOH [21]. Additional Ca^2+^ was added to get 7.4 μM free Ca^2+^ in the pipette solution, calculated using the program WEBMAXC v2.10. Rabbit IgG or M4P was added into bath solution at a concentration of 20.8 μg/ml for 30 min before recording. Ischemia/Hypoxia was induced by applying a bath solution containing 5 mM NaN_3_ and 10 mM 2-deoxyglucose (2-DG) continuously through a MicroFil (34 Gauge, WPI Inc. USA) around 10 μm away from the recording cells. The flow rate was 100 μl/min. The current–voltage relations were measured by applying voltage ramps for 250 ms from – 100 to + 100 mV at a holding potential of 0 mV. The sampling rate was 20 kHz and the filter setting was 1 KHz. Data were analysed using pClamp10, version10.2 (Molecular Devices, CA, USA).

### Statistical analysis

Data are expressed as the mean ± s.e.m. Statistical analyses were performed using GraphPad Prism version 6.0. Two-tailed unpaired student’s *t* test was used to compare two means. One-way ANOVA with Bonferroni’s multiple comparison test was used to compare ≥ 3 means. Two-way ANOVA with Bonferroni’s multiple comparison test was used to analyse motor functions and time-dependent membrane capacitance change. Mortality rates were compared using Fisher exact probability test.

## Results

### Generation and characterisation of TRPM4 blocking antibody M4P

The TRPM4 channel contains 6 transmembrane segments (T1–T6) with both N- and C-termini located intracellularly (Fig. 1a). Between transmembrane segments 5 and 6 is the 21-amino acid channel pore (P-loop). To generate the TRPM4 blocking antibody, we targeted the third extracellular region (E3) of TRPM4 channel as described previously [22]. After analysing the hydrophobicity of E3 region, we selected a 28-amino acid polypeptide from rat TRPM4 channel as antigen (Fig. 1a in the online-only Data Supplement). Of the 28 amino acids, 4 are part of the linker between T5 and the pore loop. The remaining 24 amino acids, including a glycosylation site [23], is part of the linker between the pore loop and T6. The 32-amino acid hydrophobic polypeptide including the P-loop was excluded (Fig. 1a in the online-only Data Supplement). This rat 28-amino acid polypeptide is 91.7% (22/24) homologous to the mouse TRPM4 sequence and 58.3% (14/24) homologous to the human TRPM4 sequence (Fig. 1b in the online-only Data Supplement). The antigen was tagged with a GST protein (Fig. 1c in the online-only Data Supplement) and purified for injection into the rabbit to produce a TRPM4-specific blocking antibody, named M4P in this study.

**Fig. 2:**
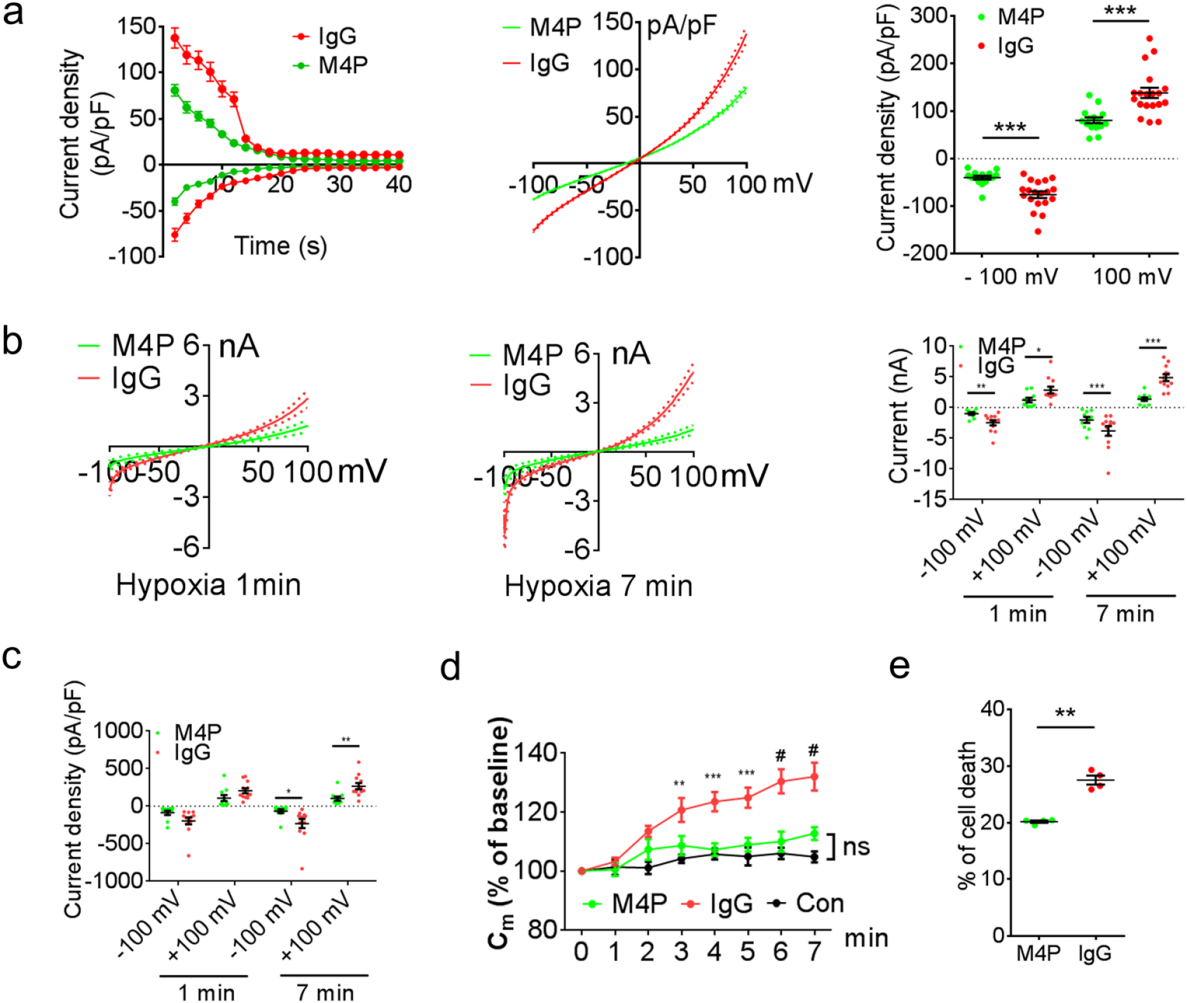
M4P blocks TRPM4 channel currents. **a** Time course of normalised currents by membrane capacitance at + 100 mV and − 100 mV from ramp protocols applied from − 100 to + 100 mV. The pipette solution contained a calculated 7.4 μM free Ca^2+^. The current-voltage relationships before desensitisation were presented as means ± s.e.m. in the middle panel. Summary of current densities at − 100 mV and 100 mV before desensitisation is shown on the right. Data were obtained from 19 cells for IgG and 15 cells for M4P. **b** Current-voltage relationship for M4P (*n* = 10) and rabbit IgG (*n* = 12) treatments from 250-ms voltage ramps after 1 min (left panel) and 7 min (middle panel) hypoxia treatment. Summary of current at − 100 mV and 100 mV under the same conditions is shown on the right. **c** Normalised current by membrane capacitance (C_m_) of M4P or rabbit IgG-treated cells after 1 min and 7 min hypoxia treatment. **d** Time course of membrane capacitance (C_m_) changes with IgG (*n* = 12) or M4P (*n* = 10) treatment under hypoxic conditions. HEK 293 cells (*n* = 13) under normoxic conditions were recorded as control. No difference was observed between M4P and control groups. **e** Summary of cell death after 24 h OGD with the treatment of M4P or IgG. *n* = 4 experiments. For **a**–**d**, TRPM4-transfected HEK 293 cells were incubated with 20.8 μg/ml IgG or M4P for 30 min before patch-clamping. In **a**, **b**, **c**, and **e**, statistical analysis was performed by two-tailed unpaired Student’s *t* test; in **d** by two-way ANOVA with Bonferroni’s post hoc analysis. *p < 0.05, **p < 0.01, ***p < 0.001, and #*p* < 0.0001. ns non-significant

**Fig. 3:**
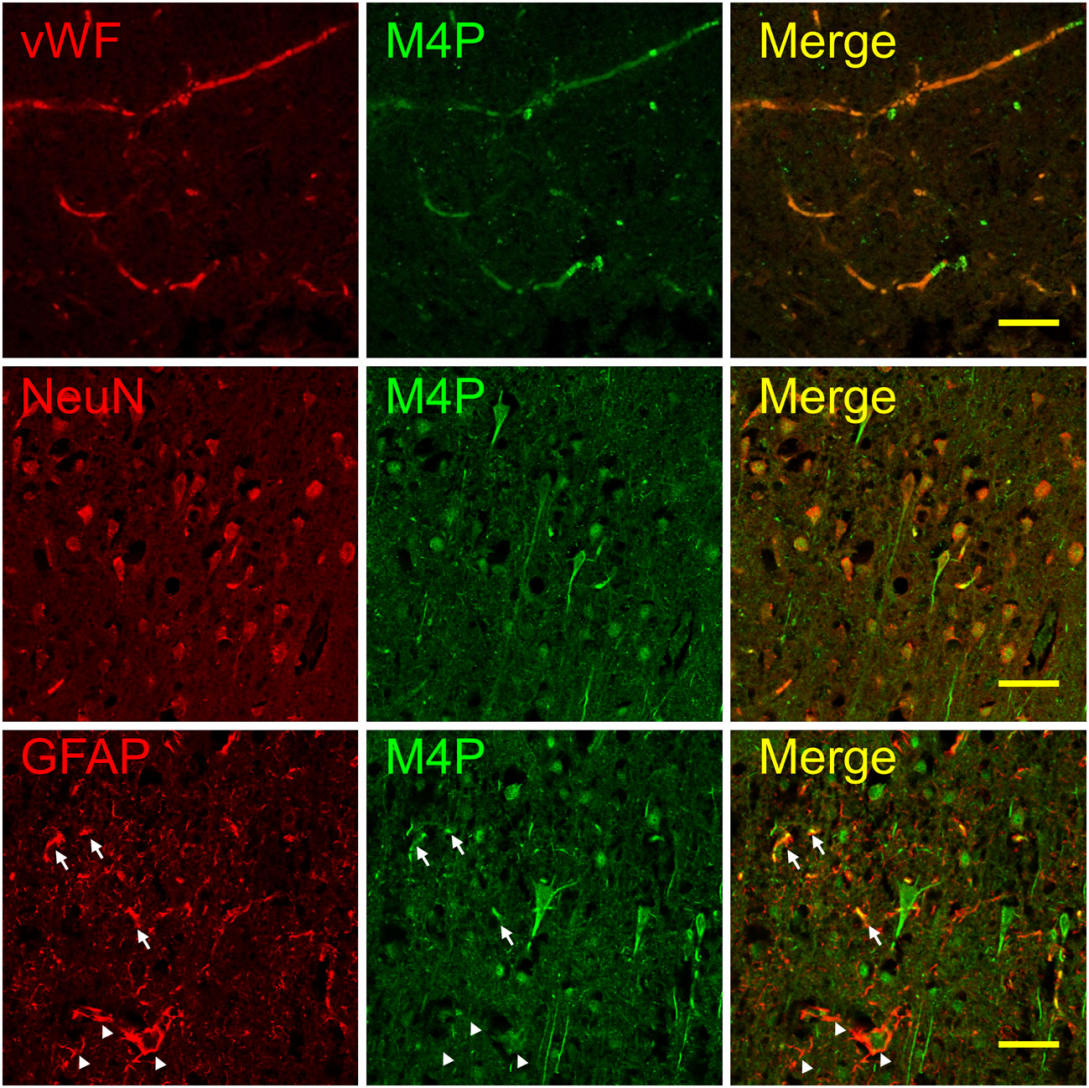
Immunofluorescent staining of brain tissues using M4P. Permanent MCAO was created in rats, and brains were collected 24 h after occlusion. In ipsilateral infarct core, M4P colocalized with vWF (upper panel). In ipsilateral penumbra region, M4P stained both cell bodies and neurites of the neurons (middle panel, arrows). Some astrocytes within the ipsilateral penumbra region were stained by M4P (arrows, lower panel), whereas other astrocytes were negatively stained (arrow heads, lower panel,). Scale bars 50 μm. Staining of contralateral hemisphere was shown in Fig. VI in the online-only Data Supplement

TRPM4 blocking antibody M4P was designed to bind to a region close to the channel pore (Fig. 1a). Using western blot, M4P successfully detected TRPM4 channel in transfected HEK 293 cells (Fig. 1b). To examine whether M4P could bind to TRPM4 channel on the surface of live cells, HEK 293 cells transfected with TRPM4 were cultured with M4P or control rabbit IgG for 30 min, 3 h, and 2 days. The cells were then fixed and a secondary antibody against rabbit IgG was applied (M4P is a rabbit polyclonal antibody). As shown in Fig. 1c, M4P could be identified on the surface of HEK 293 cells. As the incubation time increased, more cytosolic staining was observed, suggesting a translocation of M4P from cell membrane to cytosol. No surface staining was observed in cells incubated with control IgG or secondary antibody respectively (Fig. 2 in the online-only Data Supplement). To study whether the M4P treatment could regulate the expression of TRPM4 on cell membrane, surface biotinylation assay was performed. After 6 h incubation, the total TRPM4 level was not changed, whereas M4P treatment downregulated surface TRPM4 (Fig. 1d and Fig. 3a in the online-only Data Supplement). It has been reported that TRPM4 can be glycosylated [23], and the glycosylation site is located within the epitope for M4P binding (Fig. 1a, 1b in the online-only Data Supplement). We therefore compared the ratio of the fully glycosylated TRPM4 fractions with the core glycosylated TRPM4 fractions and found no change in the ratio by M4P or control IgG treatments (Fig. 3 in the online-only Data Supplement).

**Fig. 4:**
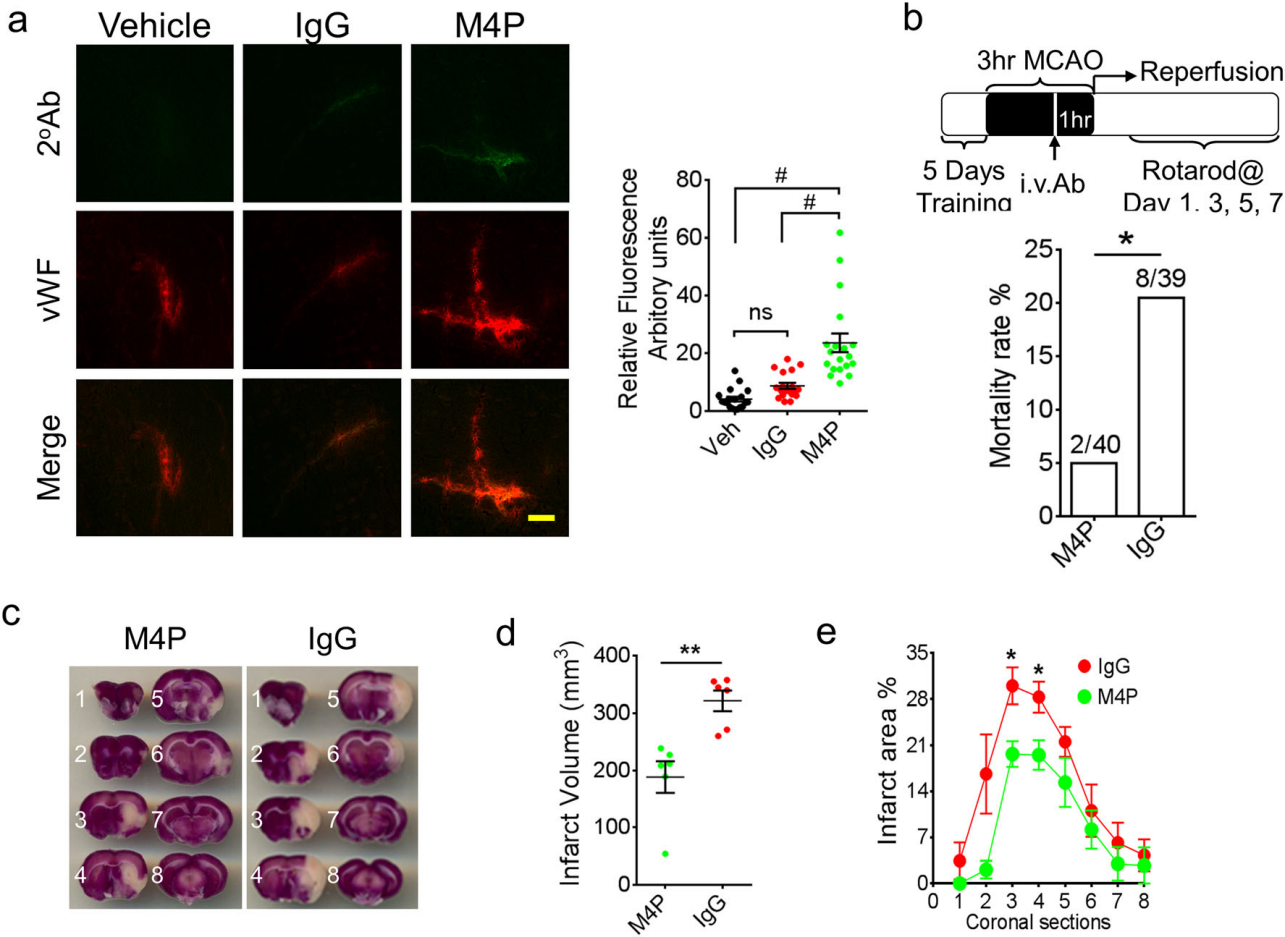
M4P application improves stroke outcome after reperfusion. **a** Immunostaining of ipsilateral hemispheres and the summary of the fluorescent intensity (19–28 images per group from 3 experiments). Vehicle, control rabbit IgG (100 μg), or M4P (100 μg) was injected 2 h after stroke induction. At 3 h, rat brains were collected and sectioned for immunostaining using secondary antibodies against rabbit IgG. Summary of the fluorescent intensity (19–28 images per group from 3 experiments) is shown on the right. **b** Experimental protocol of a 3-h MCAO followed by reperfusion. M4P (100 μg) or control rabbit IgG (100 μg) was administered intravenously 1 h prior recanalization. Mortality rates are shown (lower panel). **c** TTC staining of rat brains 1 day after operation. **d** The summary of total infarct volume (*n* = 6). **e** Section-by-section infarct area distribution (*n* = 6). In **a**, statistical analysis was performed by one-way ANOVA with Bonferroni’s post hoc analysis; in **b**, by Fisher exact probability test; in **d** and **e**, by Student’s *t* test. *p < 0.05, **p < 0.01, and #*p* < 0.0001

We further examined whether M4P could bind to the paralogous TRPM5 channel. TRPM4 and TRPM5 share only 24% homology in the 28-amino acid sequence recognised by M4P (Fig. 4a in the online-only Data Supplement). In HEK 293 cells transfected with TRPM5, M4P could not bind to TRPM5 channel as shown by immunocytochemical and western blot methods (Fig. 4b, 4c in the online-only Data Supplement), suggesting that M4P does not cross react with TRPM5.

### M4P inhibits TRPM4 current and protects transfected HEK 293 cells from hypoxia

To examine whether M4P could inhibit TRPM4 channel activity, whole-cell patch clamp was first performed on HEK 293 cells transfected with mouse TRPM4. The cells were pre-incubated with M4P or control rabbit IgG for 30 min. Under normoxic conditions, current desensitisation was observed in both M4P and control IgG treated cells (Fig. 2a left panel), similar to a previous study [24]. M4P incubation greatly inhibited TRPM4 peak current densities (Fig. 2a middle panel). At + 100 mV, the current was reduced by 41%, from 137.7 ± 10.9 pA/pF in control IgG group to 80.72 ± 6.2 pA/pF in M4P group (Fig. 2a right panel). A similar change was observed at − 100 mV. We further examined whether M4P could block TRPM4 channel under hypoxic conditions. HEK 293 cells were again incubated with M4P or control IgG for 30 min. Hypoxia was induced by applying bath solution containing 5 mM NaN_3_ and 10 mM 2-DG to the cells. As shown in Fig. 2b left panel, M4P pretreatment greatly reduced TRPM4 current 1 min after hypoxia treatment, the peak current was reduced from 2.86 ± 0.54 nA at +100 mV in IgG group to 1.23 ± 0.36 nA in M4P group, representing a 57% reduction (Fig. 2b right panel). The reductions were even more prominent at 7 min after hypoxia treatment (Fig. 2b middle panel). The peak current was reduced from 4.89 ± 0.56 nA at + 100 mV in IgG group to 1.38 ± 0.29 nA in M4P group, representing a 72% reduction. This result suggests that hypoxia could enhance TRPM4 activity. We further examined current density changes. At 7 min after hypoxia treatment, M4P incubation significantly reduced current density (pA/pF), from 266.28 ± 40.69 pA/pF at + 100 mV in IgG group to 101.83 ± 27.02 pA/pF in M4P group, representing a 62% reduction (Fig. 2c). Hypoxia treatment has been reported to increase cell volume, causing oncosis in cells expressing TRPM4 [3]. Therefore, we measured the membrane capacitance at different time points under hypoxic conditions (Fig. 2d). Membrane capacitance is known to have a positive linear correlation with cell volume [25]. After 1 min hypoxic incubation, the membrane capacitance started to increase gradually in IgG treated cells. At 7 min, the cell volume was increased to 132% of baseline at 0 min. In sharp contrast, the membrane capacitance in M4P treated cells was not significantly different from that in control cells under normoxic conditions (Fig. 2d). This result suggests that M4P could ameliorate hypoxia-induced cell swelling. The effect of M4P on cell death was further assessed in HEK 293 cells transfected with TRPM4. After 24 h oxygen glucose deprivation, cells receiving M4P treatment exhibited 20% cell death, less than that of the control IgG treatment (27.5%) (Fig. 2e).

### Detection of TRPM4 channel by M4P in ischemic stroke

**Fig. 5:**
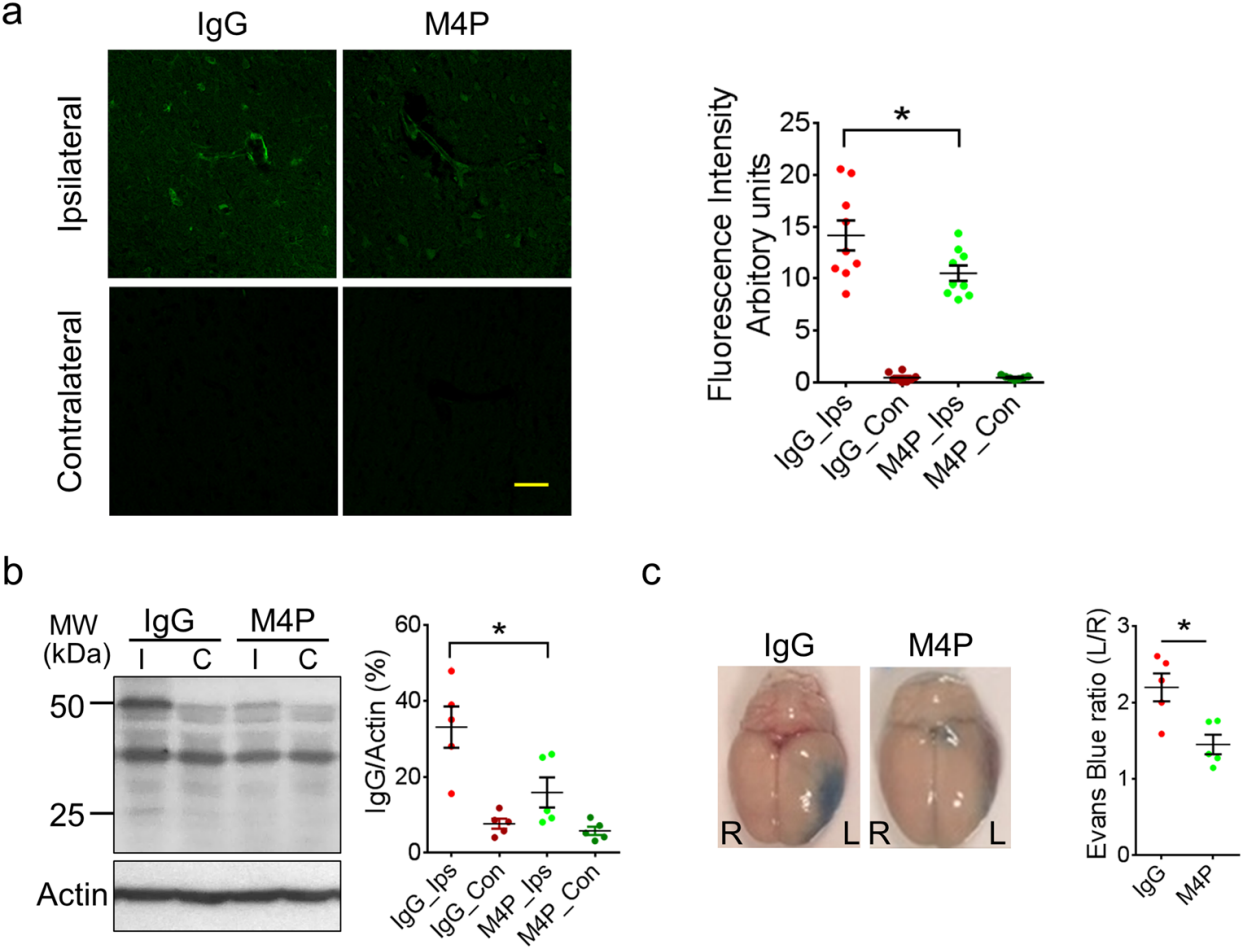
M4P improves BBB integrity after stroke reperfusion. **a** Extravasation of M4P or control rabbit IgG in rat brains. The experimental protocol is similar as in Fig. 4b. Immunostaining was performed 1 day after stroke reperfusion. Fluorescent intensities from 3 experiments were quantified on right. **b** Western blot using anti-rabbit IgG to detect antibody extravasation for control rabbit IgG and M4P rabbit polyclonal antibodies. Heavy chain (50 kDa) and light chain (25 kDa) were labelled. Antibody extravasations (both heavy and light chains) were summarised from 5 experiments (normalised to actin). **c** Evans blue extravasations were performed 1 day after stroke reperfusion to evaluate BBB integrity. The dye in the ipsilateral hemispheres (left) was normalised to that in the contralateral hemispheres (right). *n* = 5 rats/group. In **a** and **b**, statistical analysis was performed by one-way ANOVA with Bonferroni’s post hoc analysis. In **c**, statistical analysis was performed by two-tailed unpaired Student’s *t* test. *p < 0.05

**Fig. 6:**
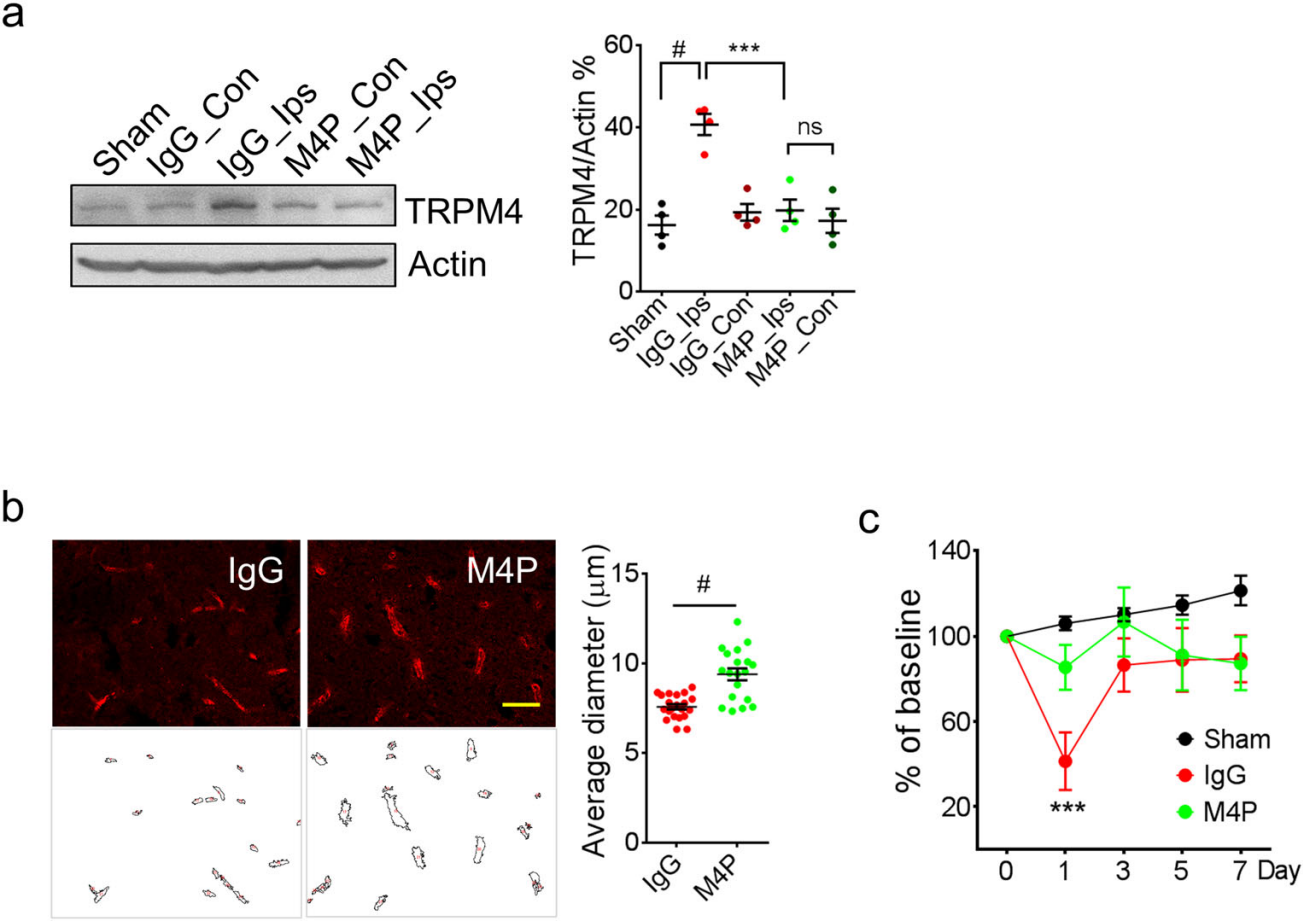
M4P treatment ameliorates vascular damage and improves functional recovery after stroke reperfusion. **a** Western blot detecting TRPM4 expression in sham-operated rats and MCAO rats treated with IgG or M4P. Tissue lysates were prepared from penumbra regions in ipsilateral hemispheres of control IgG or M4P treated MCAO rat (Ips) and symmetrical regions in contralateral hemispheres (Con), and from similar regions of sham-operated rat brain (Sham). *n* = 4. **b** The blood vessels within the ipsilateral hemispheres 1 day after reperfusion was stained by anti-vWF antibody. Feret vascular diameter was quantified and summarised (17 images per group from 3 experiments). **c** Assessment of motor functions by Rotarod test (*n* = 8). In **a**, statistical analysis was performed by one-way ANOVA with Bonferroni’s post hoc analysis; in **b**, by two-tailed unpaired Student’s *t* test; in **c**, by two-way ANOVA with Bonferroni’s post hoc analysis. ***p < 0.001, #*p* < 0.0001. ns non-significant

To examine whether M4P could bind to TRPM4 channel in stroke animal, a permanent MCAO model was created in rats. One day after occlusion, the brains were collected, sectioned, and stained with M4P and markers for endothelium, neuron, and astrocyte. M4P was shown to colocalize with endothelial marker vWF within the infarct core region where few neurons and astrocytes survived (Fig. 3 upper panel). Within the penumbra region, M4P colocalized well with NeuN on the cell bodies of neurons. Additionally, M4P could stain neurites that were negative to NeuN staining in these surviving neurons (Fig. 3 middle panel). Both neurons and blood vessels can be stained by M4P within the penumbra region (Fig. 5 in the online-only Data Supplement). For GFAP positive cells, only a portion of astrocytes were stained by M4P (Fig. 3 lower panel). In contralateral hemisphere, M4P did not stain endothelia, astrocytes, and most neurons. A small number of NeuN positive neurons were detected by M4P (Fig. 6 in the online-only Data Supplement). Similarly, very low staining of M4P was observed in the same regions of sham-operated rat brain (Fig. 7 in the online-only Data Supplement). This result demonstrated that M4P could detect TRPM4 upregulation in ischemic brain after stroke.

### M4P attenuates cerebral injury in ischemia reperfusion

As TRPM4 expression in cerebral vasculature is upregulated as early as 2 h after stroke induction [9], we hypothesise that M4P application could reduce reperfusion injury during early phase of stroke. To examine whether M4P could bind to TRPM4 channels in cerebral vasculature in stroke, we injected 100 μg of M4P, control rabbit IgG, or a vehicle solution intravenously into rats 2 h after stroke induction. The dose of antibody was optimised and selected based on a previous study [26]. The brains were collected 1 h later. Immunostaining using a secondary antibody against rabbit IgG (M4P was generated from rabbit) revealed that M4P immunolabelled TRPM4 expressed on the vascular wall within the infarct region, whereas control IgG and vehicle did not (Fig. 4a). This result indicated that M4P could bind to TRPM4 channels in cerebral vessels that were occluded. Next, we evaluated the therapeutic effect of M4P in a stroke model of early ischemia (3 h) reperfusion. In this transient MCAO model, M4P antibody was injected intravenously 2 h after stroke induction. Reperfusion was achieved by removing the filament at 3 h (Fig. 4b). In this model, the mortality rate in the M4P group (5%) was significantly lower than that in the IgG group (20.5%) (Fig. 4b). TTC staining was performed to calculate infarct volume at 1 day after operation. Comparing with the control IgG group, M4P treatment greatly reduced infarct volume. The total infarct volume was decreased from 321 mm^3^ in the control IgG group to 188 mm^3^ in the M4P group (Fig. 4c, d). Section-by-section infarct area analysis showed that the reduction was obvious at the 3rd and 4th sections (Fig. 4e)

### M4P protects cerebral vasculature in ischemia reperfusion

To evaluate the disruption of blood–brain barrier (BBB) following 3 h MCAO and reperfusion, antibody extravasation was examined 1 day after operation. The brains were perfused before sectioning to remove residual antibodies within the circulation. Immunofluorescent staining using secondary antibody against rabbit IgG revealed no antibody leakage within contralateral hemispheres in both control rabbit IgG and M4P groups, whereas in ipsilateral hemispheres, more antibody extravasation was observed in IgG group as compared with M4P group (Fig. 5a). Furthermore, we performed western blot to detect antibody extravasation. As shown in Fig. 5b, both heavy and light chains were detected. Similar to immunofluorescent staining, gel quantification verified that antibody leakage was low in contralateral hemispheres. In ipsilateral hemispheres, more antibody extravasation was observed in control IgG-treated animals (Fig. 5b). These results suggest that both IgG and M4P could pass through a disrupted BBB and enter the brain parenchyma. M4P treatment could protect vasculature, leading to a less antibody leakage. To further evaluate BBB integrity, Evans Blue dye was injected intravenously 1 day after operation. As shown in Fig. 5c, MCAO resulted in a dye leakage in the ipsilateral hemispheres. Again, M4P treatment significantly reduced Evans Blue leakage.

TRPM4 upregulation has been reported in both permanent and transient stroke models [12, 13]. In this study, TRPM4 upregulation was again detected in the penumbra region in ipsilateral hemispheres of control IgG-treated animals using western blot (Fig. 6a). Interestingly, M4P treatment successfully attenuated the increase of TRPM4 expression (Fig. 6a), further supporting that M4P incubation could downregulate TRPM4 expression. Next, we compared the vascular morphology between the IgG group and the M4P group (Fig. 6b). Vascular diameter is an indicator of vascular integrity [12]. After recanalization, a healthy vessel can be successfully perfused, and a larger diameter can be observed accordingly. Therefore, a smaller diameter indicates that the vessels are not properly perfused due to a vascular injury. After comparing the blood vessels within the infarct area and the surrounding penumbra region, it was revealed that the vascular diameter in the M4P group is significantly larger than that in the IgG group. This result suggests that M4P treatment could enhance vascular integrity after stroke reperfusion, similar to what we observed in a stroke reperfusion model using TRPM4 siRNA [12]. Rotarod test indicated that motor functions were greatly improved by M4P treatment at day 1 post stroke induction (Fig. 6c). The performance of M4P-treated animals was of no difference to sham-operated animals. Both M4P and IgG groups recovered fast after day 3. All these data support that M4P treatment could ameliorate reperfusion injury by improving vascular integrity.

## Discussion

Recently, TRP channels have attracted attention as potential therapeutic targets for stroke [27]. Here, we generated and characterised a TRPM4-specific blocking antibody M4P, targeting a region within the third extracellular domain of TRPM4 channel [28]. As the antibody-binding motif is located extracellularly, M4P has easy access to membrane TRPM4 channels. The binding of M4P to TRPM4 channel was validated by both in vitro and in vivo studies. M4P was shown to inhibit TRPM4 activity via two mechanisms. The first is to inhibit the channel directly which could be more potent under hypoxic conditions, because ATP depletion can enhance TRPM4 activity [1]. Secondly, incubation with M4P downregulated TRPM4 expression on cell surface, most likely via a mechanism seen in therapeutic antibodies in cancer treatment [29], where the formation of receptor-antibody complex induces endocytosis and subsequent protein degradation. The results from both in vitro data using cultured cells and in vivo data in stroke model indicated that M4P application could inhibit the expression of TRPM4. Importantly, as TRPM4 upregulation under hypoxia is known to cause oncotic cell death [18], our data strongly suggest that M4P incubation alleviates cell swelling under hypoxic conditions and reduces cell death.

Therapeutic antibodies targeting various receptors have been reported for stroke management [30]. We employed stroke reperfusion model to evaluate the efficacy of M4P, antibodies were delivered 1 h before filament withdrawal, mimicking clinical scenario. One challenge for this study is that whether the antibody given before recanalization could travel to the hypoxic brain region where the blood vessel is blocked. Our results showed that M4P accumulated inside the vessels within the infarct area, possibly via collateral circulation, and bind to the endothelial TRPM4 where the channel is upregulated as early as 2 h after stroke [12].

It has been reported previously that 3 h occlusion resulted in a maximum infarct formation, and the occlusion time longer than 3 h did not increase infarct volume [31]. In our 3-h stroke reperfusion model, M4P application could reduce infarct formation, accompanied with lower mortality rate and improvement of functional outcome. The results from Evans blue, antibody extravasation, and direct measurement of vascular diameter all strongly support that M4P treatment could improve vascular integrity, in line with our previous study using a similar stroke reperfusion model where cerebral oedema formation was ameliorated by TRPM4 siRNA [12].

Evidence showed that intravenous immunoglobulin (IVIG) treatment could modulate complement activation following stroke [32]. But the IgG dose (0.1 mg/250 g) used in this study is much lower than the IVIG (> 0.5 g/kg). As M4P is a therapeutic antibody, it is possible that the immunomodulation effect may contribute to its therapeutic outcome in addition to blocking TRPM4. If a higher dosage is tolerable, increase of M4P dose could enhance its modulatory effect on the immune system during stroke.

Apart from vascular protection, M4P may yield neuroprotection via blocking neuronal TRPM4 channels. TRPM4 channel has been shown to be upregulated in neurons after stroke [33]. In experimental autoimmune encephalomyelitis, TRPM4 inhibition protects neurons against neuroinflammation [34]. Here, we demonstrated that M4P could bind to neurons close to the infarct core. In healthy brain, M4P is less likely to pass through BBB. However, as ischemic insult disrupts BBB, large molecules such as antibodies can enter brain parenchyma and block neuronal TRPM4 channels. Further study is needed to evaluate whether M4P yields a direct neuroprotection on neurons.

Besides stroke therapy, M4P can serve as a research tool to study TRPM4 channel. As the antigenic epitope for M4P production is unique to TRPM4 channel, M4P is more specific than other blockers. Glibenclamide inhibits TRPM4 via SUR1 which requires the surface expression of SUR1 protein with a high ratio to TRPM4 [4, 35]. Also, neurons express SUR1-K_ATP_ channel complex [36], which can be blocked by glibenclamide. Another TRPM4 blocker 9-phenanthrol interacts with TMEM16A channel, affecting vascular contraction [37]. In heart, 9-phenanthrol non-selectively inhibits transient outward, rapid delayed rectifier, and inward rectifier K^+^ currents [21]. Furthermore, as a member of polycyclic aromatic hydrocarbons, the toxicity of 9-phenanthrol remains a concern for in vivo usage.

In conclusion, we have developed a TRPM4-specific blocking antibody that can alleviate stroke injury during reperfusion. This strategy could avoid contraindications arising from other TRPM4 blockers. A future humanised TRPM4 blocking antibody could be a potential therapy for stroke in humans.

### Contributions statement

All authors contributed to the study conception and design. SWL and YG prepared the reagents. BC and GN performed the surgery. SWL and YG carried out functional and biochemical studies. SW performed electrophysiological experiments. BC, SWL, YG, SW, and PL analysed the data.

## Electronic supplementary material

ESM 1 (DOCX 24584 kb)
