# Supplementary material for: TRPM4-specific blocking antibody attenuates reperfusion injury in a rat model of stroke
Source: Pflugers Arch. 2019 Oct 29;471(11):1455–66. doi: 10.1007/s00424-019-02326-8 (PMC6892354; doi:10.1007/s00424-019-02326-8)
Supplement: Supplementary file 1 — (DOCX 24584 kb) [file 424_2019_2326_MOESM1_ESM.docx]

**SUPPLEMENTAL MATERIAL**

TRPM4 specific blocking antibody attenuates reperfusion injury in a rat model of stroke

Cover Title: TRPM4 blocking antibody in stroke

Bo Chen^1^* PhD, Yahui Gao^1^* BA, Shunhui Wei^1^* PhD, See Wee Low^1^ BA, Gandi Ng^1^ BA, Dejie Yu^2^ BA, Tian Ming Tu^3^ MD, PhD, Tuck Wah Soong^2^ PhD, Bernd Nilius^4^ MD, PhD, Ping Liao^1,5,6^^†^ MD, PhD

^1^Calcium Signalling Laboratory, Department of Research, National Neuroscience Institute, Singapore, ^2^Department of Physiology, Yong Loo Lin School of Medicine, National University of Singapore, ^3^Department of Neurology, National Neuroscience Institute, Singapore, ^4^Department of Cellular and Molecular Medicine, KU Leuven, Leuven, Belgium, ^5^Duke-NUS Medical School, Singapore, ^6^Health and Social Sciences, Singapore Institute of Technology, Singapore.

* These authors contribute equally in this study.

^†^ Correspondence to Ping Liao, MD, PhD. Calcium Signalling Lab, National Neuroscience Institute, 11 Jalan Tan Tock Seng, Singapore 308433. E-mail: [ping_liao@nni.com.sg](mailto:ping_liao@nni.com.sg) Fax: (65) 6256 9178; Tel: (65) 6357 7611.

**Supplemental**

**Figure I**

**a**

QDRSSNCSAERGSWAHPEGPVAGSCVSQ

LPSILRRVFYRPYLQIFGQIPQEEMDVALMNP

*****

**b**

**Rattus norvegicus** **E G I L R P Q D R S L P S I L R R V F Y R P Y** **L Q**

**Mus musculus E G I L R P Q D R S L P S I L R R V F Y R P Y L Q**

**Homo sapiens**  **E G L L R P R D S D F P S I L R R V F Y R P Y L Q**

**Rattus norvegicus I F G Q I P Q E E M D V A L M N P S N C S A E R G**

**Mus musculus I F G Q I P Q E E M D V A L M I P G N C S M E R G**

**Homo sapiens I F G Q I P Q E D M D V A L M E H S N C S S E P G**

**Rattus norvegicus S W A H P E G P V A G S C V S Q Y A N W**

**Mus musculus S W A H P E G P V A G S C V S Q Y A N W**

**Homo sapiens F W A H P P G A Q A G T C V S Q Y A N W**

*

**c**

**1**


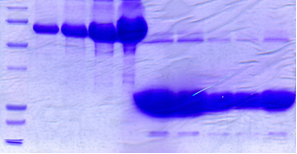


**BSA (µg)**

**2**

**5**

**GST-M4P elution**

**10**

GST-M4P

75

**MW**

**(kDa)**

50

37

25

**1**

**2**

**3**

**4**

**5**

**M**

**M**

**M**

**Supplemental Fig. I Generation and characterization of antibody M4P (a)** A 28-amino acid polypeptide between transmembrane segment 5 and 6 from the rat TRPM4 channel was synthesized for M4P production. The 32-amino acid hydrophobic polypeptide spanning across the pore lining sequence was excluded. Asterisk: N-glycosylation site. Channel pore: boxed in green. **(b)** Alignment of the linker sequences between transmembrane segments 5 and 6 of TRPM4 from *Rattus norvegicus, Mus musculus, and Homo sapiens*. The rat 28 amino acid sequences for generating M4P were circled with boxes. The pore loops were labelled in filled grey boxes. Asterisk: N-glycosylation site. **(c)** Coomassie Blue Staining of a SDS-PAGE gel showing the expression of GST-fused 28 amino acid rat TRPM4 polypeptide for immunization.

**Supplemental Figure II**


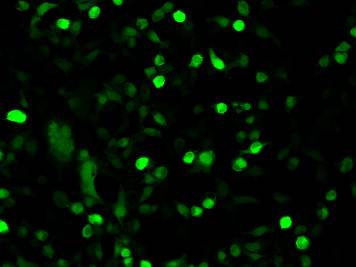

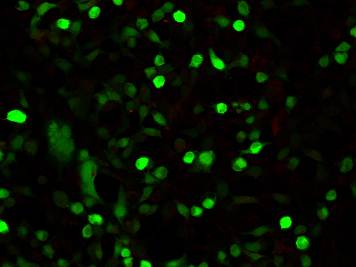

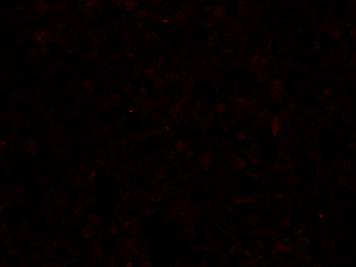


**2^o^ Ab**


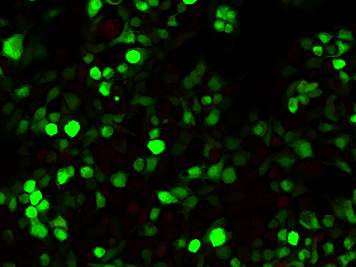

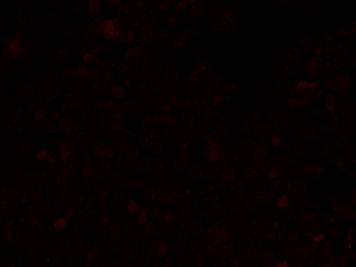

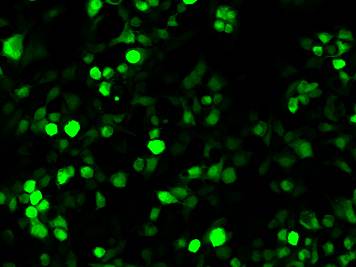


**IgG**

**TRPM4**

**TRPM4**

**Merge**

**Merge**

**Supplemental Fig. II** **Both secondary antibody alone treatment and rabbit IgG treatment fail to stain TRPM4 channels.** HEK 293 cells transfected with TRPM4 were incubated with secondary antibody (upper panel) or rabbit IgG (lower panel) for 6 hrs. The cells were then fixed and processed for immunostaining. Scale bar: 50 µm

**Supplemental Figure III**

**a**


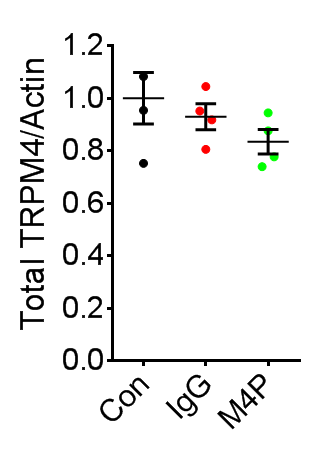


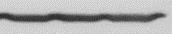


Actin

Total


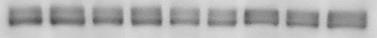


TRPM4_H

TRPM4_L

Con

IgG

M4P

**b**


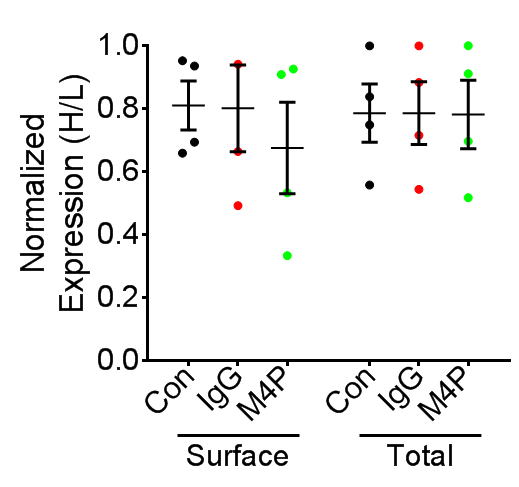


**Supplemental Fig. III** **M4P treatment does not change total glycosylated TRPM4** (**a)** Detection and quantification of total TRPM4 channel expression. (**b)** Quantification of ratio of fully glycosylated TRPM4 (TRPM4_H) fractions to core glycosylated TRPM4 (TRPM4_L) fractions. TRPM4_H indicated protein fully glycosylation. TRPM4_L indicated core glycosylation. TfR: transferrin receptor. TRPM4 transfected HEK 293 cells were incubated with rabbit IgG (1.3 µg/ml) and M4P (1.3 µg/ml) for 6 hrs. Con: control group received no treatment. n=4 experiments for (a), (b), and statistical analysis was performed by One Way ANOVA with Bonferroni’s post hoc analysis. In (a), F (2,9) = 1.464, *p* = 0.2815. In (b), surface ratio, *p* = 0.7005; total ratio, *p* = 0.9993.

**Supplemental Figure IV**

**a**

**Rat TRPM4**  **E G I L R P Q D R S L P S I L R R V F Y R P Y** **L Q**

**Rat TRPM5**  **Q A L L H P H D G R L E W I F R R V L Y R P Y L Q**

**Rat TRPM4 I F G Q I P Q E E M D V A L M N P S N C S A E R G**

**Rat TRPM5 I F G Q I P L D E I D E A R V - - - N C S - - - -**

**Rat TRPM4 S W A H P - E G P V A G S C V S Q Y A N W**

**Rat TRPM5 - - L H P L L L D S S A S C P N L Y A N W**

*

**MW**

**(kDa)**

TRPM5

GFP

**c**

**b**

GFP

NT


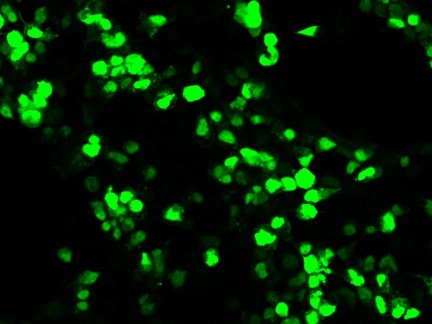

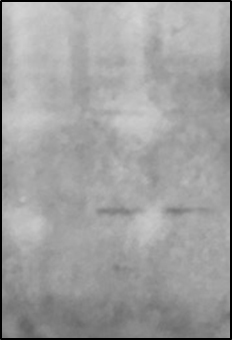


TRPM5/M4P

150

75

M4P

50

37


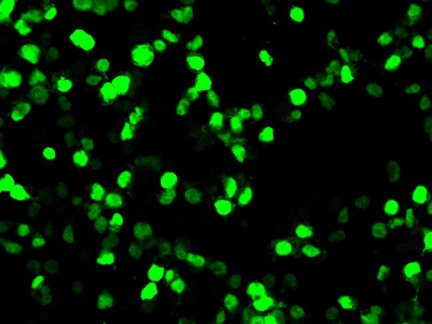


GFP


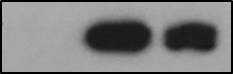


GFP


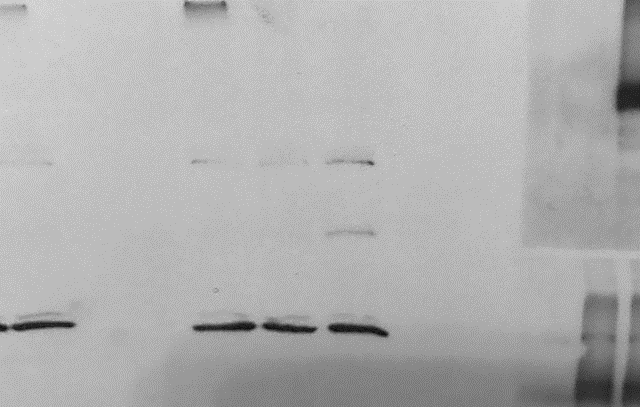


TRPM5


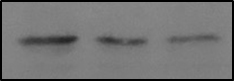


Actin

**Supplemental Fig. IV** **M4P does not bind to TRPM5 channel** **(a)** Alignment of the linker sequences between transmembrane segments 5 and 6 of TRPM4 from *Rattus norvegicus* TRPM4 channel (NP_001129701.1) and TRPM5 channel (NP_001178825.1). Asterisk: N-glycosylation site. The polypeptides used for generating M4P were circled with boxes, and TRPM4 and TRPM5 share only 24% homology (7/29). The pore loops were labelled in filled grey boxes, and TRPM4 and TRPM5 share 62% homology (13/21) in this region. **(b)** HEK 293 cells were transfected with pIRES EGFP/TRPM5 and pEGFP. M4P could not detect TRPM5 channel (upper panel). **(c)** Western blot using M4P could not detect TRPM5 channel which is around 130 kD. Control GFP protein and TRPM5 protein were successfully detected by commercial antibodies. NT: non-transfected.

**Supplemental Figure V**


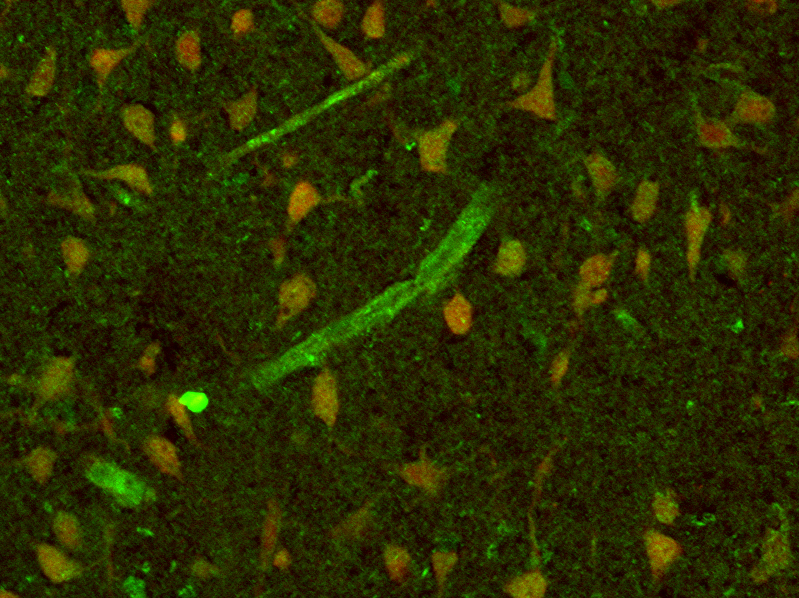

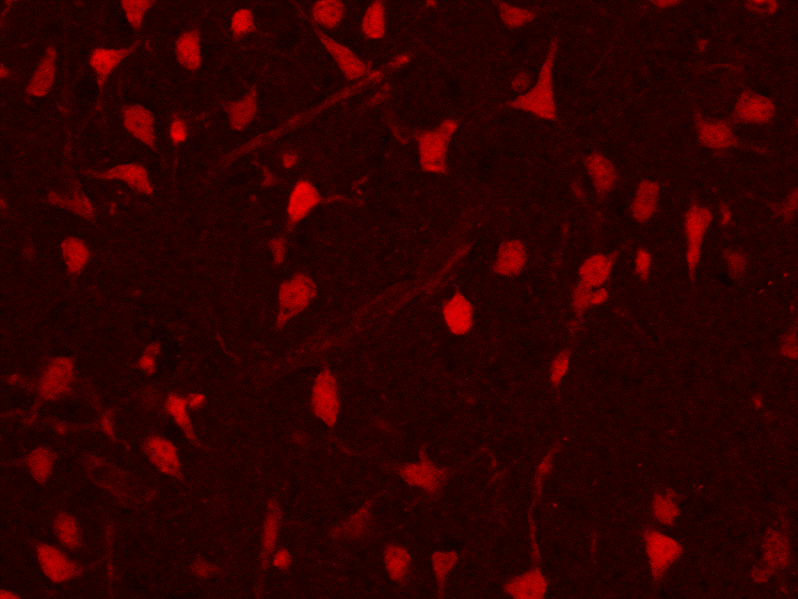

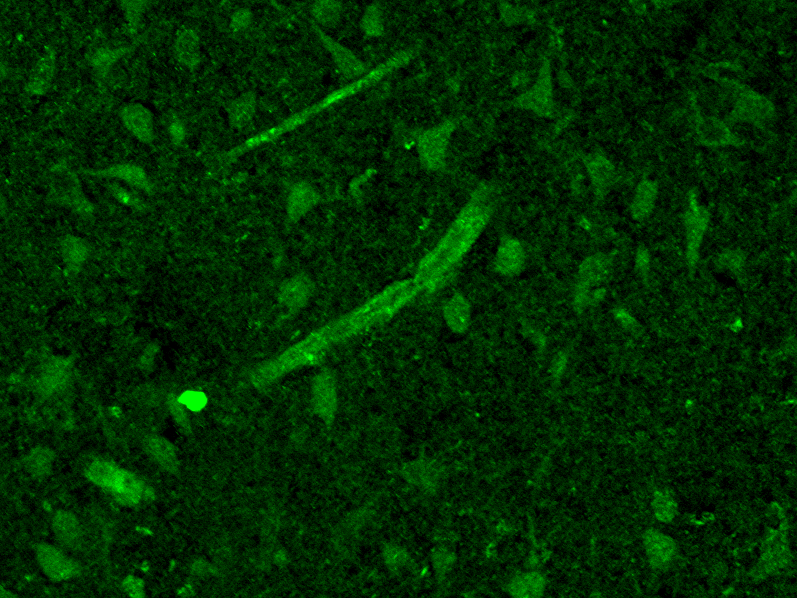


NeuN

M4P

Merge

**Supplemental Fig. V M4P stain with NeuN in penumbra region of ipsilateral hemisphere**. Co-staining of TRPM4 using M4P antibody with anti-NeuN in ipsilateral hemisphere of rat brains 1 day after MCAO. Scale bar: 50 µm.

**Supplemental Figure VI**


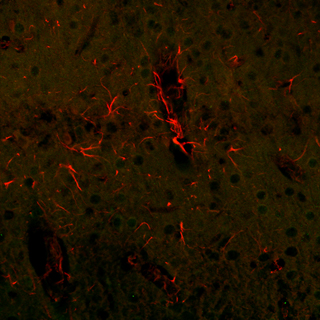

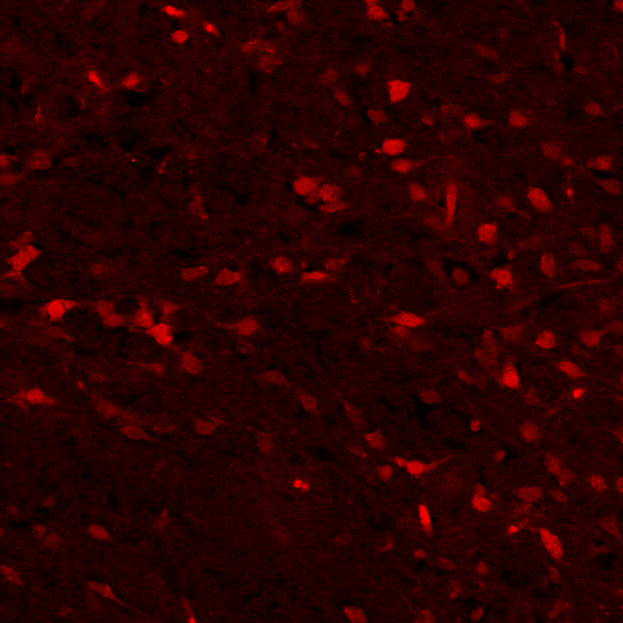

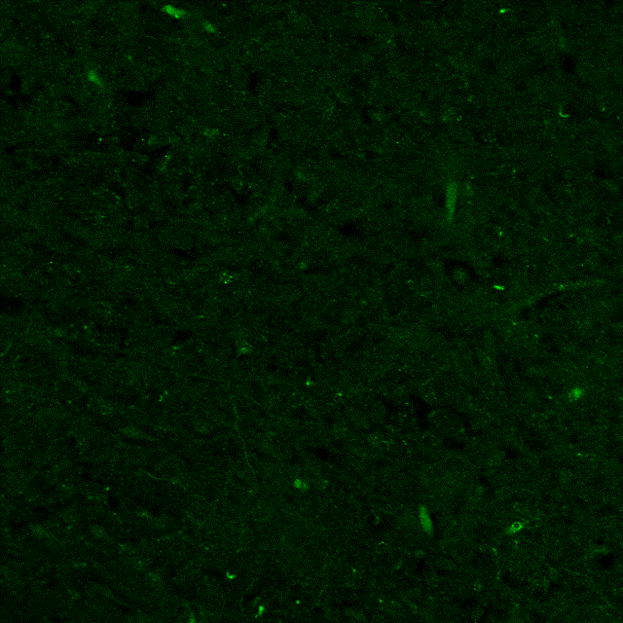

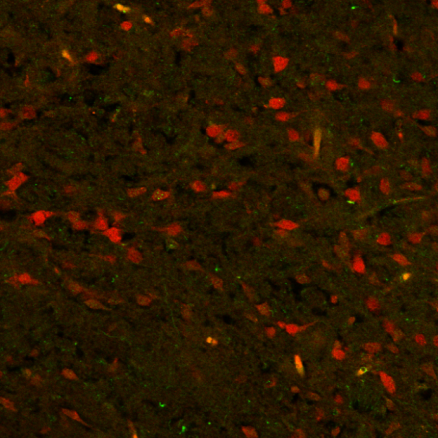

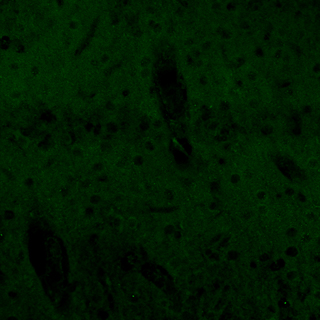


M4P

Merge


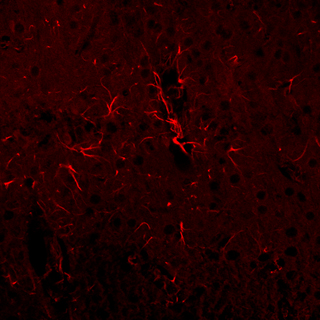


GFAP


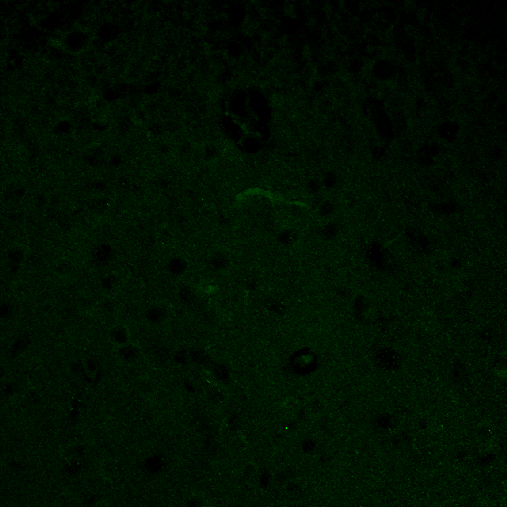

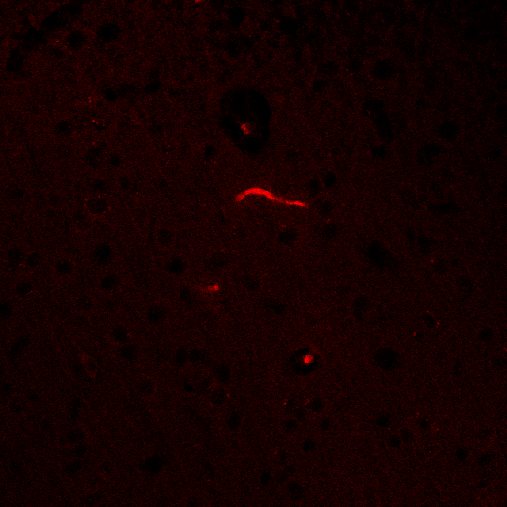

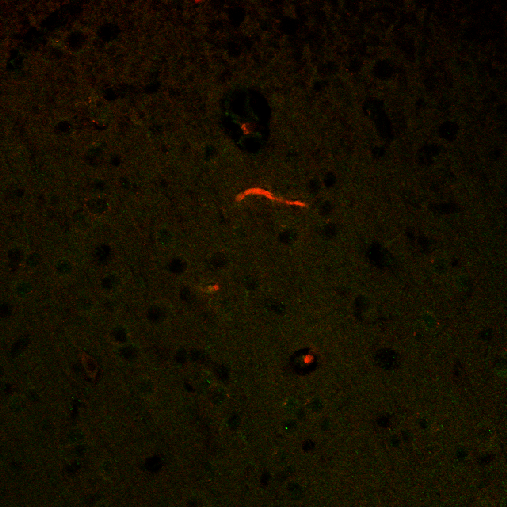


NeuN

M4P

Merge

vWF

M4P

Merge

**Supplemental Fig. VI M4P does not stain astrocytes and most neurons in contralateral hemisphere**. Co-staining of TRPM4 using M4P antibody with anti-vWF, anti-NeuN and anti-GFAP in contralateral hemisphere of rat brains 1 day after MCAO. Arrows: co-localization of M4P with NeuN in neurons. Scale bar: 50 µm.

**Supplemental Figure VII**


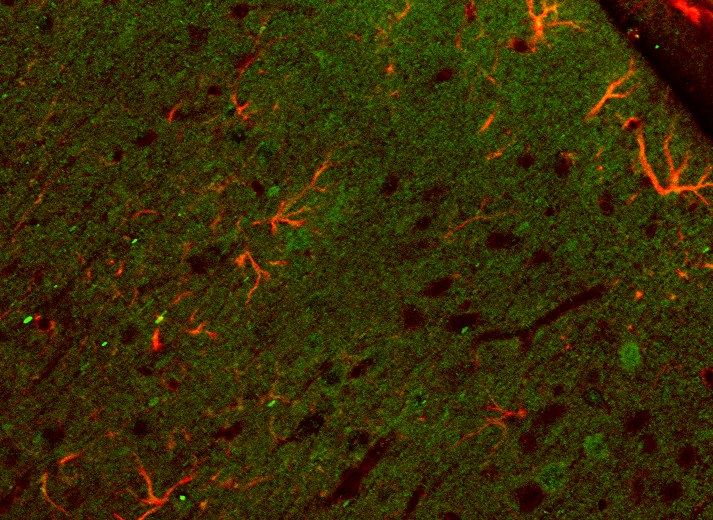

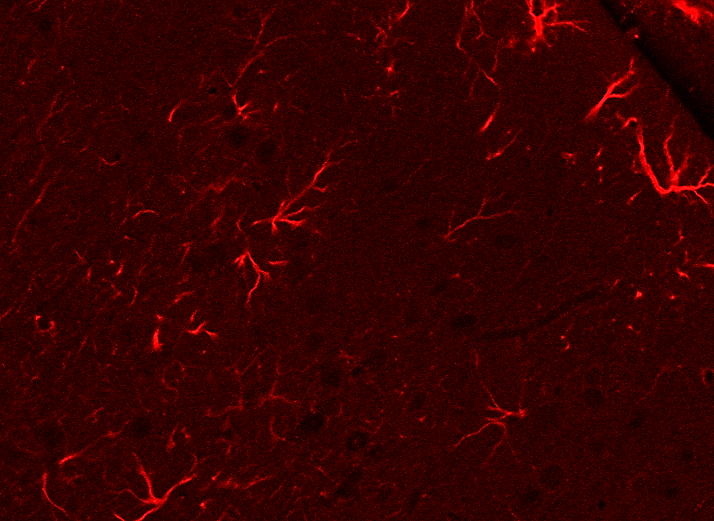

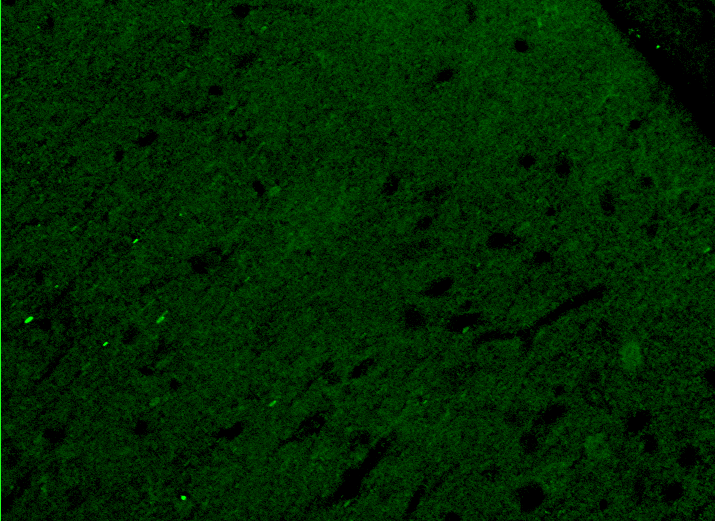


GFAP

M4P

Merge


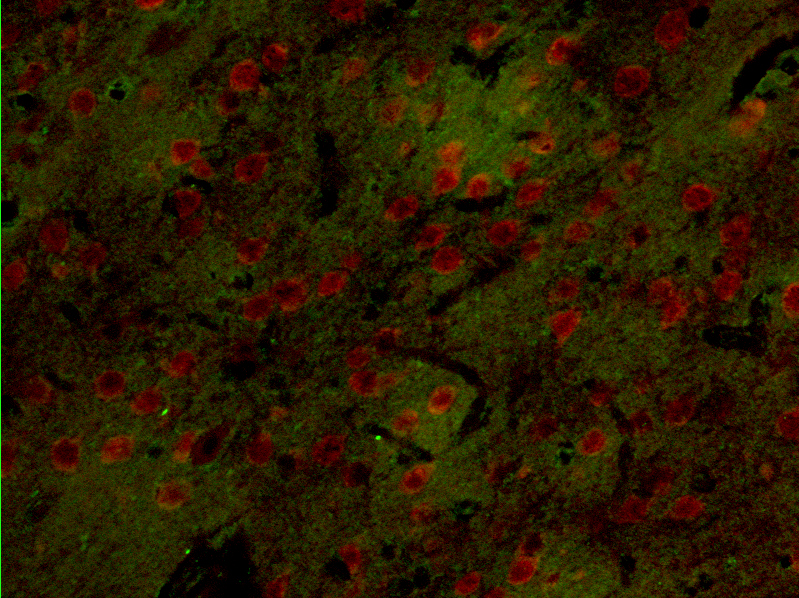

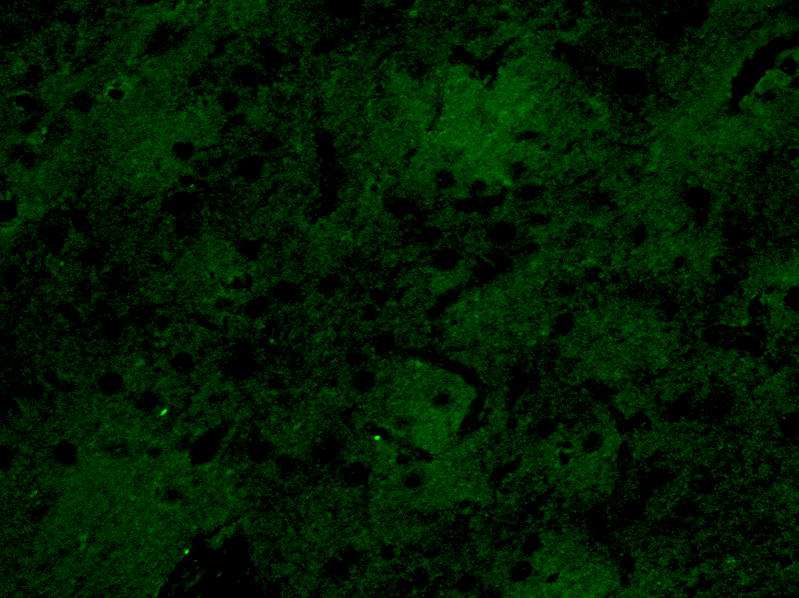


M4P

Merge


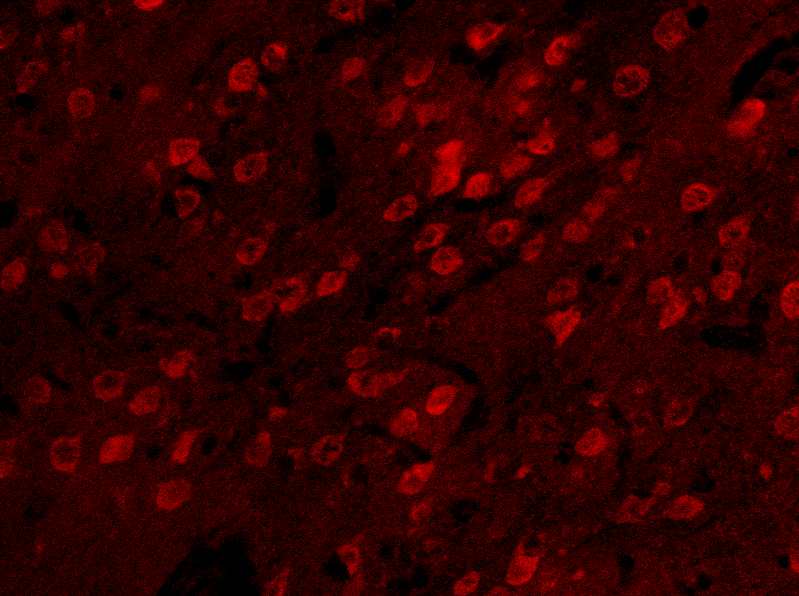


NeuN


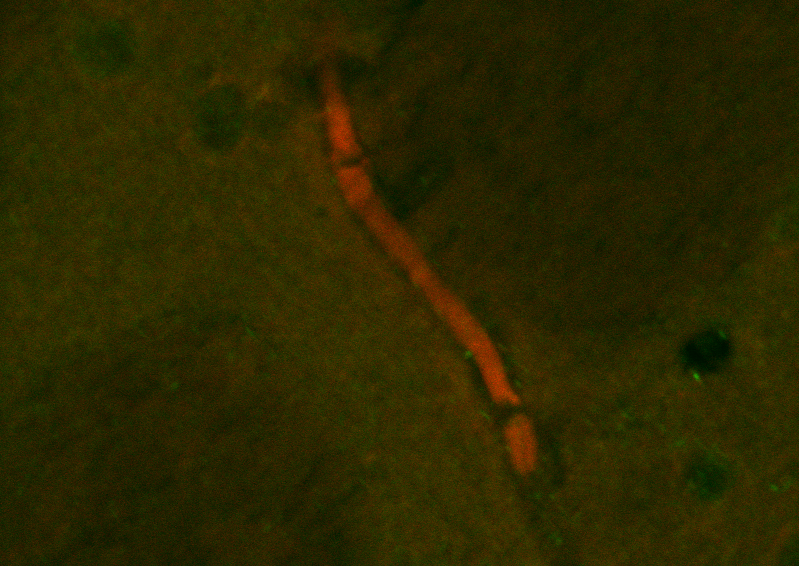

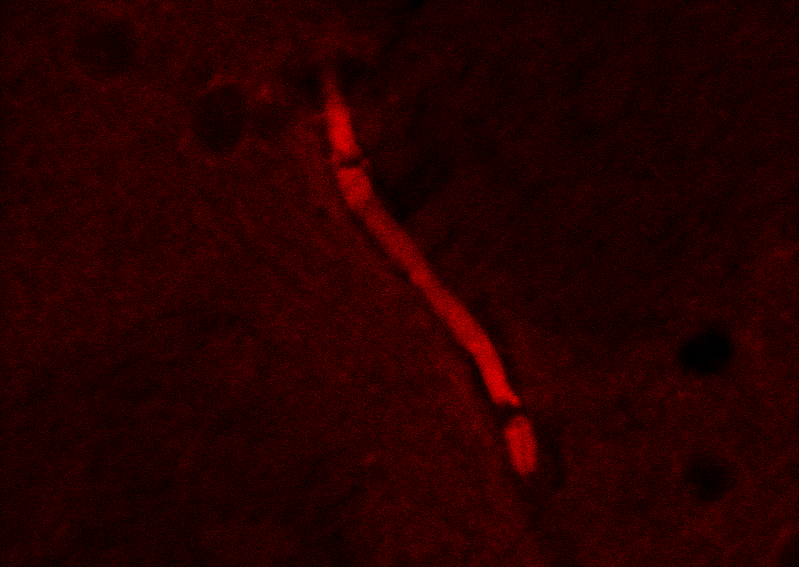

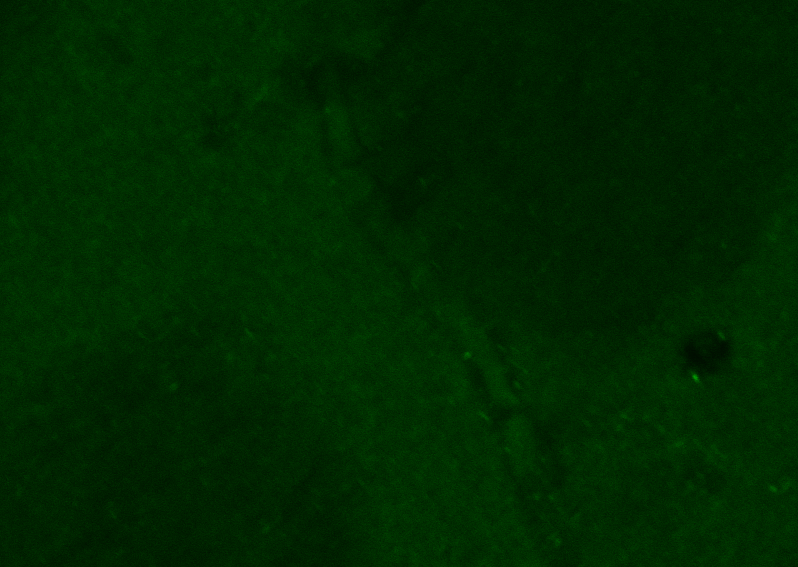


vWF

M4P

Merge

**Supplemental Fig. VII Immunostaining using M4P in sham-operated rat brain**. Co-staining of TRPM4 using M4P antibody with anti-vWF, anti-NeuN and anti-GFAP in same regions as Fig. VI in sham-operated rat brains. Scale bar: 50 µm.
